# The Nervous Complications of Influenza

**Published:** 1900-10-06

**Authors:** 


### THE NERVOUS COMPLICATIONS OF INFLUENZA.

The discussion at the recent meeting of the British Medical Association on influenza as it affects the nervous system tended to show how widespread are the effects of this sometimes trivial but often terrible disease. Dr. Judson Bury, who introduced the discussion, after showing that whatever part of the frame might seem to bear the brunt of the attack, the nervous system almost always suffered, pointed out that the cases of nerve affection might be broadly separated into two groups. In the first group, he said, we place nervous diseases which develop during or shortly after the febrile stage, and are sometimes the sole representatives of the effects of the influenza poison. Meningitis and hæmorrhagic encephalitis are the best examples of this group, and we may assume—indeed, we have proof—that in these diseases brain tissue is directly attacked by the bacillus.

In the second group we place nervous diseases which usually occur after the attack has subsided; neurasthenia and multiple neuritis are good examples of this sequence of events. Here we assume that the toxins produced by the bacilli are more dilute, less virulent than in the first group. Sometimes they are sufficiently powerful to initiate degeneration in nerve tissue, whereas in other cases they appear to be incapable of setting up definite histological changes, their power being limited to a disturbance of the functions of the nervous system.

Dr. Judson Bury and Dr. Buzzard were quite at one in regard to the extraordinary variety of nervous symptoms which may be met with among the sequelæ of influenza.

A tendency was shown by some of the speakers to regard some of the nervous affections which are met with after attacks of influenza as due to some pre-existing disturbance or weakness of the nervous system laid bare or brought again into activity by the depressing influence of the disease; but Dr. Buzzard pointed out that, although this might account for a certain proportion of the nerve diseases observed as sequelæ of influenza, it by no means held good for the large majority of cases. Nor was it necessary to call up such a hypothesis. It was impossible to believe that the pain in the back and bones, the aching head and eyeballs, which would characterise attacks affecting a large number of persons simultaneously in a community, did not represent the effect of a toxin upon previously healthy structures; and if this was certain, as he thought was the case, he failed to see any difficulty in equally ascribing the later nerve diseases which followed influenza to the direct effect of a toxin also. Dr. Buzzard especially insisted on the widespread and bizarre distribution of the symptoms in the nervous diseases which sometimes follow influenza, as pointing to their originating from the direct effect of a toxin upon healthy nervous structure. In describing such disease it was impossible, he said, to cramp and confine our nomenclature to such narrow cabins as "encephalitis," "neuritis," "myelitis," "poliomyelitis," "meningitis," and the like, for in many of the cases the influence of the toxin had evidently been extensive, embracing widely separated neurons, and such as it was impossible to include in any recognised category of diseases of the nervous system.

Finally, he expressed the opinion that the relative as well as the absolute number of grave diseases of the nervous system ascribable to toxæmia had very largely increased during the last few years, and said that he was disposed to think that, although this increase was by no means to be attributed exclusively to the invasion of influenza (for he felt sure that there were many other as yet unlabelled toxins at work), yet that a large proportion must be credited to the direct influence of the disease under consideration.

